# Mitochondrial sphingosine-1-phosphate lyase is essential for phosphatidylethanolamine synthesis and survival of *Trypanosoma brucei*

**DOI:** 10.1038/s41598-020-65248-x

**Published:** 2020-05-19

**Authors:** Ladan Dawoody Nejad, Michael Stumpe, Monika Rauch, Andrew Hemphill, Roger Schneiter, Peter Bütikofer, Mauro Serricchio

**Affiliations:** 1 Institute of Biochemistry and Molecular Medicine, University of Bern, Bern, Switzerland; 2 Graduate School for Cellular and Biomedical Sciences, University of Bern, Bern, Switzerland; 3 Division of Biochemistry, Department of Biology, University of Fribourg, Fribourg, Switzerland; 4 Institute of Parasitology, Vetsuisse Faculty, University of Bern, Bern, Switzerland

**Keywords:** Lipidomics, Lipids, Proteins, Optical imaging, Cell growth, Mitochondria

## Abstract

Sphingosine-1-phosphate is a signaling molecule involved in the control of cell migration, differentiation, survival and other physiological processes. This sphingolipid metabolite can be degraded by the action of sphingosine-1-phosphate lyase (SPL) to form hexadecenal and ethanolamine phosphate. The importance of SPL-mediated ethanolamine phosphate formation has been characterized in only few cell types. We show that in the protozoan parasite *Trypanosoma brucei*, expression of TbSpl is essential for cell survival. Ablation of TbSpl expression increased sphingosine-1-phosphate levels and reduced *de novo* formation and steady-state levels of the glycerophospholipid phosphatidylethanolamine (PE). Growth of TbSpl-depleted parasites could be in part rescued by ethanolamine supplementation to the growth medium, indicating that the main function of TbSpl is to provide ethanolamine phosphate for PE synthesis. In contrast to most cell types analyzed, where SPL localizes to the endoplasmic reticulum, we found by high-resolution microscopy that TbSpl is a mitochondrial protein. In spite of its mitochondrial localization, TbSpl depletion had no apparent effect on mitochondrial morphology but resulted in aggregation of acidocalcisomes. Our results link mitochondria to sphingolipid metabolism and suggest possible roles for PE in acidocalcisome function.

## Introduction

Sphingosine-1-phosphate (S1P) is a sphingolipid metabolite involved in the control of cell migration, differentiation and survival, as well as numerous other (patho-)physiological processes (reviewed in ^1–5^). S1P levels are regulated through the actions of three enzyme activities: i) sphingosine kinase synthesizes S1P by phosphorylating the long chain base sphingosine, ii) sphingosine phosphohydrolases catalyze the reverse reaction to produce sphingosine, and iii) sphingosine-1-phosphate lyase (SPL) irreversibly degrades S1P by cleaving the acyl chain between carbon atoms 2 and 3, resulting in the formation of hexadecenal and ethanolamine phosphate (reviewed in ^3,6^). Ethanolamine phosphate, in turn, may represent a substrate for phosphatidylethanolamine (PE) synthesis by the CDP-ethanolamine branch of the Kennedy pathway^7^. SPL is dependent on pyridoxal 5′-phosphate as cofactor for enzymatic activity^8^. In mammals and yeast, SPL is membrane-bound via a single N-terminal transmembrane domain, which is not strictly required for *in vitro* enzyme activity^9,10^. In contrast, at least in *Saccharomyces cerevisiae*, the transmembrane domain is necessary for SPL activity *in vivo* and may be involved in oligomer formation^10^. The structure has been solved for bacterial SPL homologues^11–13^ and soluble forms of human^9,14^ and yeast^11^ SPL.

Cytosolically produced S1P can be secreted into the extracellular environment via specific transporters (reviewed in^6^), where it binds to cell surface G protein-coupled receptors, known in mammals as S1PR_1–5_ (reviewed by^15–17^), to induce numerous cellular and tissue-specific responses. Alternatively, S1P can act as intracellular second messenger to regulate processes including endosomal processing, gene expression and mitochondrial assembly and function (reviewed by^18^).

Early studies to identify the intracellular location of SPL involved sub-cellular fractionation and enzyme activity measurements. In rat liver, the highest activity of SPL was reported in microsomal and mitochondrial fractions, with the inner mitochondrial membrane sub-fraction containing most of the mitochondrial activity^8^. However, in a subsequent study, rat liver SPL was reported to associate almost exclusively with vesicles enriched in typical microsomal marker enzymes, with its active site facing the cytosol^19^. All subsequent attempts to localize SPL were carried out with tagged SPL enzymes (over-) expressed in various mammalian cells ^20,21^ and *S. cerevisiae*^10^ and reported SPL to be an endoplasmic reticulum (ER)-resident protein. One notable exception is SPL from *Legionella pneumophila*, which when expressed in mammalian cells, localized mainly to mitochondria^22^. Recently, this observation was questioned^12^, adding to the controversy about the subcellular localization of SPL.

Deletion of *Sgpl1* genes, encoding SPL in mice, resulted in developmental abnormalities in multiple organs and death of animals after 3–4 weeks after birth ^23,24^. In addition, reduced or lack of SPL activity was shown to affect normal immune function, whereas upregulation of SPL activity was reported in several human pathologic conditions ^4,25,26^. In model organisms, such as *Dictyostelium*, *Drosophila* and *Caenorhabditis elegans*, reduced or deficient SPL expression caused numerous cellular abnormalities, including dysregulation in sphingolipid metabolism. Moreover, SPL seems to play an important role in skeletal muscle repair through activation of muscle stem cells (reviewed in^5^).

In the human pathogenic parasite *Leishmania major*, deletion of genes encoding SPL resulted in reduced stationary phase differentiation and virulence^27^. In addition, knock-out parasites showed reduced production of PE and phosphatidylcholine (PC). Since the cellular and biochemical alterations could be reversed upon supplementation of parasites with exogenous ethanolamine, the report proposed that the main function of SPL in *Leishmania* is not to degrade S1P but to produce ethanolamine phosphate for glycerophospholipid synthesis^27^.

We now studied the function of SPL in *Trypanosoma brucei*, the protozoan parasite causing human African sleeping sickness and nagana in domestic animals, by generating inducible *T. brucei* SPL (TbSpl) knock-out parasites. This allowed us, for the first time, to investigate possible effects of ablation of TbSpl expression on phospholipid metabolism and viability in a time-dependent manner rather than in a null mutant which may have adapted to the loss of SPL during selection. In addition, by adding a tag to conditionally express a catalytically active TbSpl, we were able to study its subcellular localization. Our results showed that ablation of TbSpl in *T. brucei* procyclic forms caused a reduction in PE levels, leading to parasite death in culture. The growth defect could partially be rescued by addition of ethanolamine to the culture medium. Unexpectedly, confocal and high-resolution fluorescence microscopy localized TbSpl to the mitochondrion.

## Results

### Identification of TbSpl and generation of inducible knockout parasites

**Figure 1:**
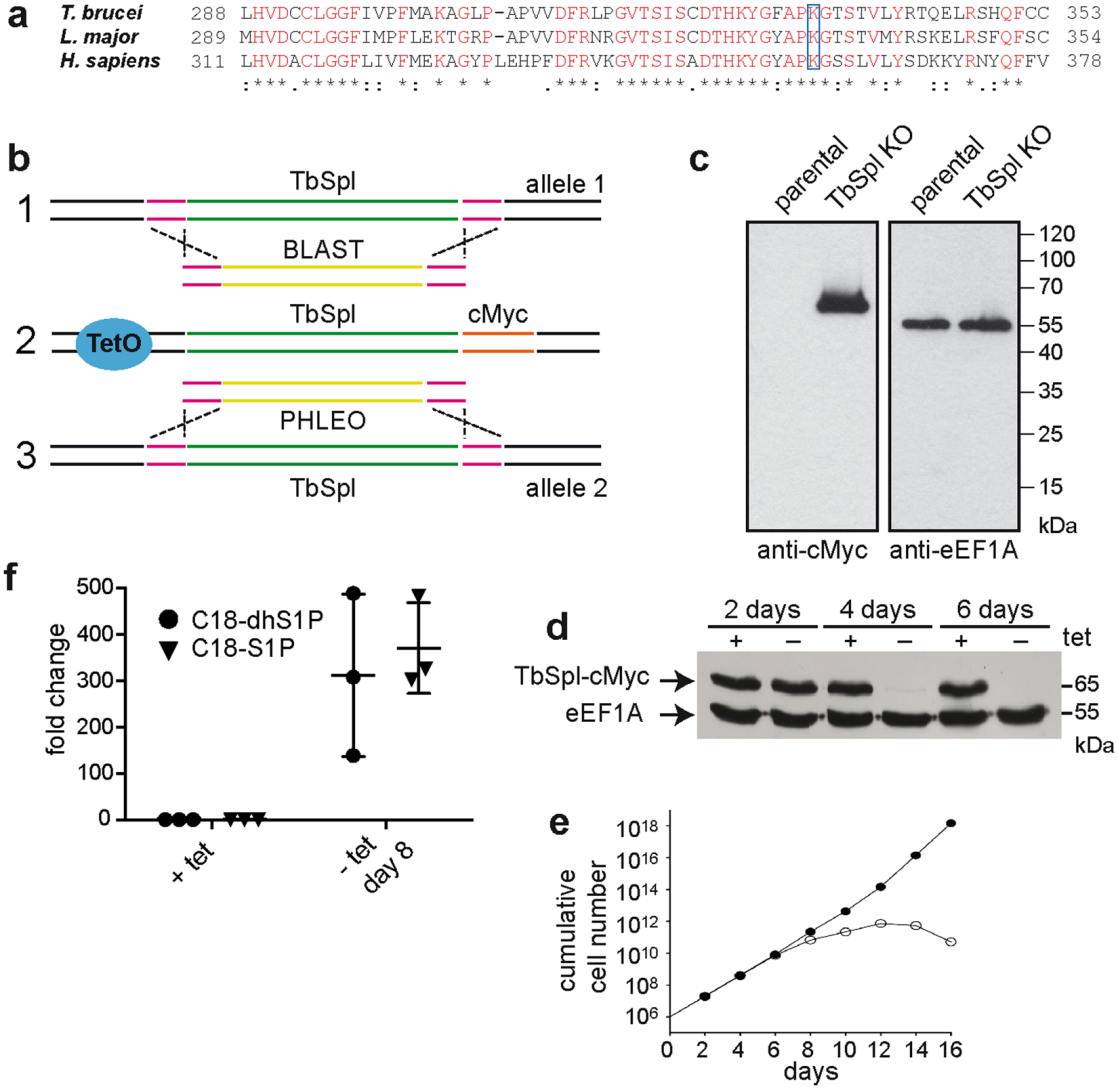
Generation of inducible TbSpl-knockout cells. (**a**) Partial sequence alignment of *T. brucei*, *L. major* and *H. sapiens* sphingosine-1-phosphate lyase sequences. Conserved amino acids are shown in red, the boxed lysine residues show the conserved catalytic site. (**b**) Strategy to generate conditional knockout parasites. First, the ORF of TbSpl from one allele was replaced with a blasticidin (BLAST) resistance gene (1). After antibiotic selection and PCR confirmation of correct replacement, a tetracycline-inducible c-myc-tagged ectopic copy of TbSpl was inserted into the genome (2), then the second allele of TbSpl was replaced with a phleomycin (PHLEO) resistance gene (3). (**c**) Immunoblot analysis of whole cell lysates from parental cells and TbSpl KO parasites expressing TbSpl-cMyc. eEF1A was used as a loading control. (**d**) Immunoblot analysis of whole cell lysates. Cells were cultured in the presence (+) or absence (−) of tetracycline for 2, 4 or 6 days and proteins detected by Western blotting. The positions of TbSpl-cMyc and eEF1A are indicated. (**e**) Growth of conditional TbSpl KO cells cultured in the presence (•) or absence (o) of tetracycline. (**f**) Relative quantification of cellular S1P levels from TbSpl KO cells cultured in presence (+) or absence (−) of TbSpl expression for 8 days. Levels of C18-S1P and C18-dhS1P (dihydrosphingosine-1-phosphate) were quantified. Individual data points (+/−SD) of fold increases of three independent determinations are shown.

The *T. brucei* genome contains a single gene (Tb927.6.3630) encoding a candidate TbSpl^28^. The deduced amino acid sequence shows 64% identity to previously characterized *L. major* SPL and 35% identity to *Homo sapiens* SPL. The predicted TbSpl protein consists of 538 amino acids with a calculated molecular mass of 59 kDa and contains the conserved pyridoxal-dependent decarboxylase domain (amino acids 138–421) found in other eukaryotic SPL proteins. The conserved lysine residue K328 in TbSpl corresponds to lysine K353 in the active site of human SPL^9^ (Fig. 1a). Secondary structure prediction programs (Phobius (31) and TMHMM (32)) identified a single N-terminal transmembrane domain between amino acids 17 and 37.

The function of TbSpl was studied by generating conditional TbSpl-knockout parasites (TbSpl KO) that express tetracycline-inducible ectopic C-terminally cMyc-tagged TbSpl (TbSpl-cMyc) (Fig. 1b). Replacement of the two TbSpl alleles in TbSpl KO parasites was verified by Southern blotting using a DNA probe hybridizing to the 3′UTR of Tb927.6.3630 (Fig. S1a). The results confirm replacement of the TbSpl open-reading frames by blasticidin and phleomycin resistance cassettes. The two antibiotic resistance genes have similar lengths; therefore, the respective bands co-migrate at ~3.5 kbp. In addition, correct integration of the antibiotic resistance genes in TbSpl KO parasites was verified by PCR using specific primers (Fig. S1b).

Expression of TbSpl-cMyc in TbSpl KO parasites cultured in the presence of tetracycline was analyzed by SDS-PAGE and immunoblotting and revealed a band at ~65 kDa (Fig. 1c). Subsequently, ablation of TbSpl-cMyc expression was studied by culturing parasites in the absence of tetracycline. Analysis by SDS-PAGE/immunoblotting revealed that TbSpl-cMyc was still present after 2 days of culture, however, the protein was no longer detectable after extended culture times (4 or 6 days) (Fig. 1d), demonstrating time-dependent depletion of TbSpl-cMyc. To determine if TbSpl expression is essential for survival of *T. brucei*, growth of TbSpl KO parasites cultured in the presence or absence of tetracycline was compared (Fig. 1e). The data show that ablation of TbSpl-cMyc expression resulted in reduced parasite growth after 6 days, followed by growth arrest and cell death after continued culture (Fig. 1e). These results demonstrate that *TbSpl* is an essential gene for survival of *T. brucei* procyclic forms in culture and that parasite death can be prevented by expressing an inducible and functionally active TbSpl-cMyc protein.

Ablation of TbSpl activity is expected to result in accumulation of cellular S1P levels. Quantification of S1P by mass spectrometry using externally added C17-S1P revealed a 300-fold increase in S1P levels in TbSpl KO parasites after depletion of TbSpl-cMyc for 8 days compared to control cells (Fig. 1f). This finding, together with the high degree of sequence identity to SPL enzymes from other organisms, is consistent with Tb927.6.3630 encoding TbSpl.

### TbSpl is localized in the mitochondrion

**Figure 2:**
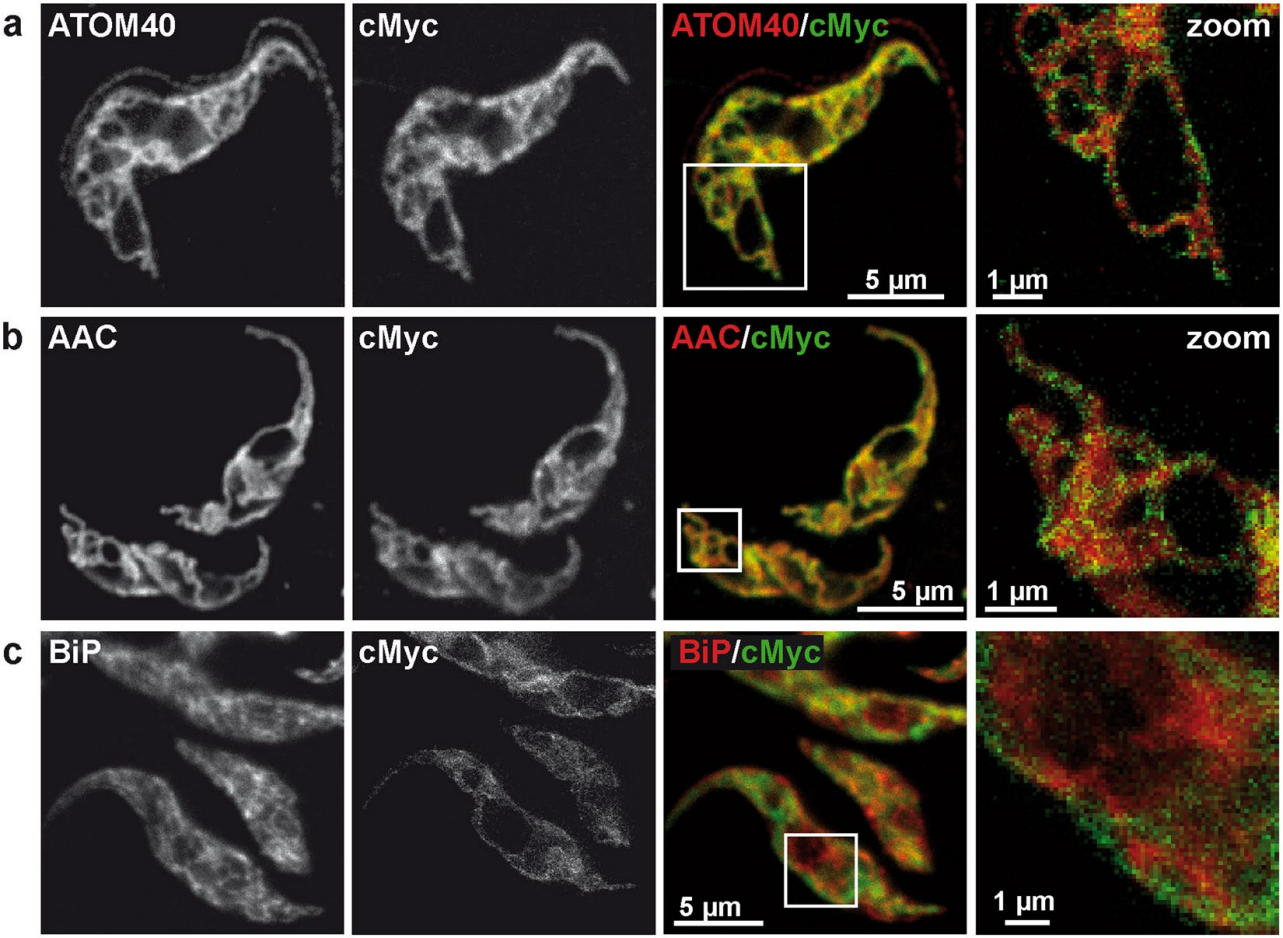
Subcellular localization of TbSpl by super-resolution microscopy. TbSpl KO cells expressing TbSpl-cMyc were fixed and processed for immunofluorescence microscopy. Cells were stained for cMyc in combination with (**a**) the −outer mitochondrial membrane protein ATOM40, (**b**) the inner mitochondrial membrane protein AAC (ADP/ATP carrier) or (**c**) the ER marker BiP. Flagellar staining by ATOM40 in (**a**) is due to unspecific staining by the ATOM40 antibody. Merged confocal images are shown in color. Zoomed-in regions show STED images. Scale bars: 5 μm in confocal, 1 μm in zoomed region.

**Figure 3:**
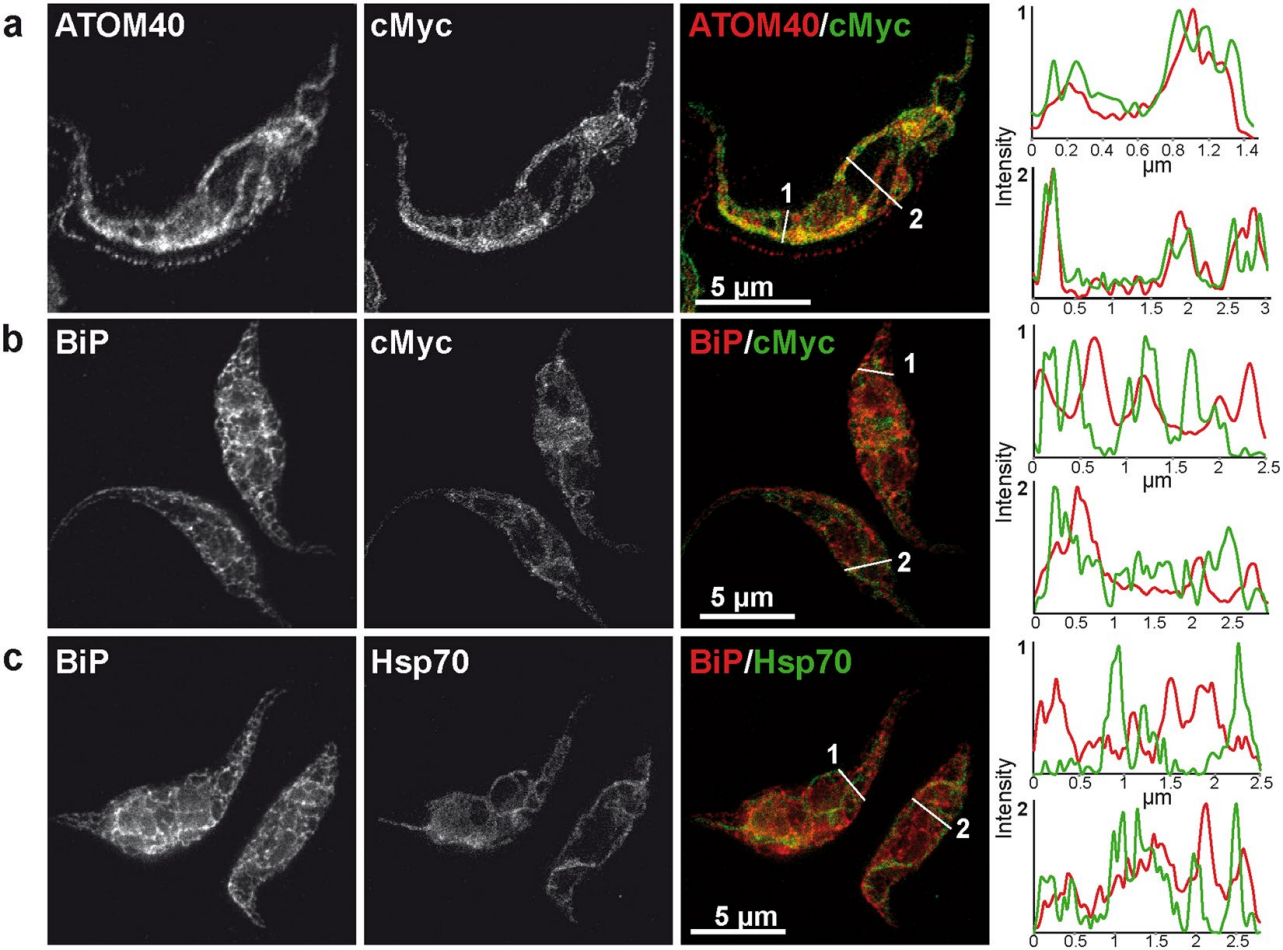
STED microscopy for TbSpl localization. STED microscopy of TbSpl KO cells stained for (**a**) cMyc and the mitochondrial outer membrane protein ATOM40, (**b**) the ER marker BiP and cMyc, and (**c**) BiP and mitochondrial Hsp70. Intensity profiles of the two individual channels for the lines in the merged images are shown on the right. Scale bars: 5 μm.

The availability of a functionally active tagged Spl protein prompted us to closely examine the subcellular localization of TbSpl using confocal immunofluorescence and STED microscopy. Analysis of parasites using anti-cMyc antibody revealed a reticular staining for TbSpl-cMyc that overlapped with the staining pattern for ATOM40, a member of the outer mitochondrial membrane protein translocation complex^29^ (Fig. 2a). A similar pattern was observed when TbSpl-cMyc was co-stained with an antibody against the ADP/ATP carrier of the inner mitochondrial membrane^30^ (Fig. 2b). In contrast, no co-localization was observed between TbSpl-cMyc and BiP, a typical marker protein for the ER^31^ (Fig. 2c). Interestingly, staining of TbSpl was not uniform along the mitochondrial tubules but appeared patchy and clustered at specific sites. In addition, using STED microscopy, we compared intensity profiles across parasites co-stained for cMyc and ATOM40 or BiP. The results show that fluorescence intensity profiles for cMyc resembled the profiles for ATOM40 (Fig. 3a). In contrast, the intensity profiles of parasites co-stained for cMyc and BiP showed little overlap (Fig. 3b) and resembled those obtained for cells co-stained for mitochondrial Hsp70 and BiP, i.e. two proteins expected to show no co-localization (Fig. 3c). Together, these results clearly identify TbSpl as a mitochondrial protein in *T. brucei* procyclic forms. The non-uniform staining suggests localization of TbSpl in mitochondrial microdomains.

### **Depletion of TbSpl affects*de novo*PE synthesis**

**Figure 4:**
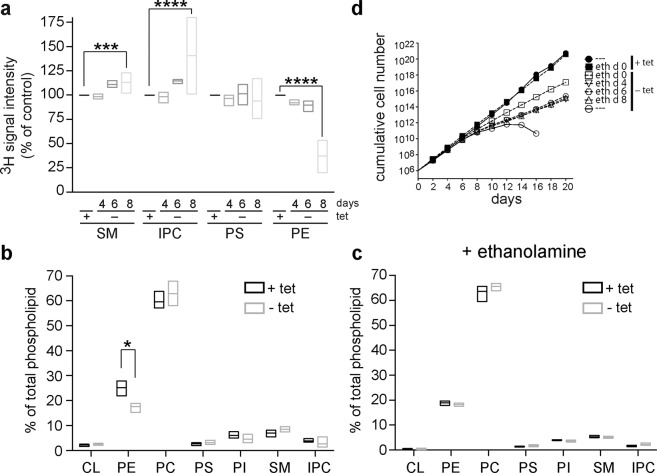
Alterations in phospholipid levels after depletion of TbSpl expression. (**a**) Analysis of *de novo* phospholipid synthesis. TbSpl knockout parasites were cultured in the presence or absence of tetracycline for a total of 4, 6, and 8 days and labeled with [^3^H]-serine for the last 24 h. Lipids were extracted, separated by one-dimensional TLC, and quantified by radioisotope scanning. Box plots of mean values (horizontal line) and the min-max range from three independent experiments are shown. ***p < 0.001; ****p < 0.0001. (**b**) and (**c**) Analysis of total glycero- and sphingophospholipid levels. Phospholipids were quantified by phosphorus determination after two-dimensional thin layer chromatography. In (**c**), parasites were treated with 50 µM ethanolamine starting from day 0 of TbSpl depletion. The data represent box plots of mean values (horizontal line) and min-max ranges from at least three independent experiments. The asterisks indicate significant differences (p < 0.05). (**d**) TbSpl KO parasites were cultured in the absence (−tet) or presence (+tet) of tetracycline. 50 μM ethanolamine was added to the medium starting from day 0, 4, 6 or 8 as indicated in the legend. The data represent mean values from two independent experiments.

Possible effects of TbSpl depletion on synthesis and steady-state levels of phospholipids were studied in TbSpl KO parasites cultured in the presence or absence of tetracycline. *De novo* synthesis of phospholipids was investigated by labeling parasites for 24 h with [^3^H]-serine followed by extraction of phospholipids and analysis by one-dimensional thin-layer chromatography (TLC) and radioisotope scanning. Representative traces of [^3^H]-serine-labeled lipids extracted from parasites after 0, 4, 6 and 8 days of TbSpl depletion are shown in Fig. S2 and demonstrate incorporation of radioactivity into sphingomyelin (SM), inositolphosphoryl ceramide (IPC), phosphatidylserine (PS) and PE. Similar results have been reported before^32^. Quantification of individual peaks revealed that the amounts of radioactivity in SM and IPC increased after depletion of TbSpl for 8 days compared to control cells. In contrast, ablation of TbSpl expression resulted in a reduction in radiolabeled PE (Fig. 4a). In addition, analysis of steady-state phospholipid levels using two-dimensional TLC and lipid phosphorus quantification showed decreased levels of PE (<70% of control levels) in parasites depleted of TbSpl for 8 days (Fig. 4b). No significant changes were seen in all other phospholipid classes, except for a (likely compensatory) increase in PC. These results demonstrate that ablation of TbSpl expression results in decreased production and steady-state levels of PE and increased formation of SM and IPC.

### Ethanolamine supplementation rescues PE deficiency and reduced growth of TbSpl-depleted parasites

In *L. major*, Spl is required for PE synthesis by providing ethanolamine phosphate as substrate for PE formation by the Kennedy pathway^27^. If this function was conserved in *T. brucei*, supplementation of exogenous ethanolamine should rescue the defect in PE synthesis and restore growth of *T. brucei* procyclic forms during TbSpl depletion. Indeed, quantification of PE levels revealed that the addition of ethanolamine to the culture medium prevented the drop in PE levels during depletion of TbSpl (Fig. 4c). In addition, when ethanolamine was added to the culture medium at different times after induction of TbSpl depletion (days 0, 4, 6 or 8), parasites continued to divide, albeit not at the same rate as control cells (Fig. 4d). Together, these results indicate that TbSpl’s main function in *T. brucei* procyclic forms is to provide ethanolamine phosphate for PE production.

### TbSpl depletion affects acidocalcisomes

**Figure 5:**
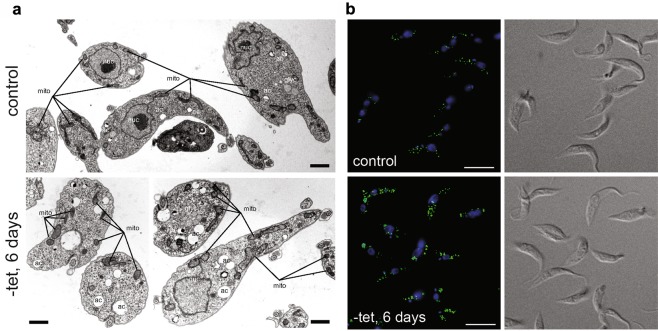
Transmission electron microscopy and acidocalcisome staining. (**a**) TbSpl KO parasites cultured in presence (+tet) or absence of tetracycline for 6 days (−tet) were processed for electron microscopy. mito = mitochondrion; ac = acidocalcisome; nuc = nucleus; glyc = glycosome. Scale bars = 0.5 µm. (**b**) Acidocalcisomes of control and TbSpl-depleted parasites were visualized with the anti-TbVP1 antibody (green), DNA was stained with DAPI (blue). Scale bars: 10 µm.

Because of its mitochondrial localization, we next investigated if depletion of TbSpl may affect functional and/or structural integrity of the mitochondrion. As markers for functional integrity, we stained TbSpl KO cells after ablation of TbSpl expression with the mitochondrial membrane potential-dependent dye MitoTracker red and measured the mitochondrial membrane potential using tetramethylrhodamine ethyl ester (TMRE). The results showed no structural defects in MitoTracker-stained mitochondria after 6 or 8 days (Fig. S3a) and no alteration in mitochondrial membrane potential after 6 days, but a trend toward hyperpolarisation after 8 days of TbSpl depletion (Fig. S3b). Similarly, using transmission electron microscopy we detected no morphological changes in mitochondria after depletion of TbSpl (Figs. 5a, S4). However, TbSpl-depleted parasites showed an accumulation of enlarged acidocalcisomes (Fig. 5a). This observation was confirmed by fluorescence microscopy using an antibody against TbVp1, a marker for acidocalcisomes^33^, showing markedly enlarged acidocalcisomes after TbSpl depletion when compared to control parasites (Fig. 5b).

## Discussion

The canonical role of SPL is to irreversibly degrade S1P, a sphingolipid signaling molecule with pleiotropic extra- and intracellular functions. In contrast, relatively little is known about the role of SPL in the formation of hexadecenal and ethanolamine phosphate, the products of S1P degradation by SPL. While hexadecenal has been shown to sensitize mitochondria for BAK activation^34^ and apoptosis^35^, ethanolamine phosphate is generally thought to feed into the PE branch of the Kennedy pathway. We now show that inhibition of ethanolamine phosphate formation in *T. brucei* by depletion of TbSpl expression leads to reduced *de novo* synthesis and steady-state levels of PE, ultimately resulting in parasite death. These effects could be prevented by exogenous supply of ethanolamine during TbSpl ablation, demonstrating that parasite death is directly linked to the reduced availability of ethanolamine phosphate rather than the elevated levels of S1P in TbSpl KO parasites.

A similar observation has been made before in *Leishmania* after deletion of the gene encoding *L. major* Spl^27^. Besides increased levels of the upstream metabolites sphingosine and S1P, *Leishmania* KO parasites showed defects in differentiation and decreased cell viability in stationary phase, which could be reversed by addition of ethanolamine to the culture medium^27^. The reason for a more severe effect of Spl depletion in *T. brucei* compared to Leishmania may be due to differences in the relative contribution of Spl-mediated ethanolamine production for PE synthesis via the Kennedy pathway in the two parasites, differences in the importance of the Kennedy pathway for PE synthesis altogether, or a different role of sphingosine-1-phosphate in signaling. More recently, neural-specific ablation of SPL in mouse brain was shown to decrease brain PE levels, resulting in impaired autophagy. Again, these effects could be reversed by providing PE to the growth medium of cultured neurons derived from SPL KO mice^36^. Together, these observations indicate that, at least in certain cells and tissues, the primary function of SPL may not be to control S1P levels and S1P-mediated signaling, but to provide ethanolamine phosphate for PE synthesis. This pathway may be particularly important in cells in which PE synthesis by the Kennedy pathway is essential for proliferation and survival, as in *T. brucei* procyclic and bloodstream forms ^32,37,38^(reviewed in^39^).

Our observation that ectopically expressed C-terminally cMyc-tagged TbSpl was able to complement endogenous TbSpl showed that the tag did not interfere with its function. More importantly, expression of a tagged functional enzyme allowed us to study its intracellular localization by immunofluorescence/STED microscopy. Co-staining with organelle-specific marker proteins revealed that TbSpl is localized to the mitochondrion. This observation is consistent with a recent report in which TbSpl was identified as a member of the *T. brucei* mitochondrial outer membrane proteome^40^. In contrast, based on studies in rat and human cells SPL is generally considered an ER protein^19–21^. However, these studies were carried out by overexpressing tagged SPL proteins with unknown enzymatic activity. In addition, localization was examined with diffraction-limited microscopy, which often lacks the required resolution to distinguish between ER and mitochondrial membranes. Based on our findings and the results from earlier subcellular fractionation experiments showing dual localization of SPL activity in both mitochondrial and ER fractions^8^, more detailed analyses are clearly required to precisely localize SPL in mammalian cells. Interestingly, STED microscopy showed TbSpl was not uniformly localized in the mitochondrial membrane. It is tempting to speculate that these sites of TbSpl enrichment may represent contact sites between the ER and the mitochondrial outer membrane, a sub-compartment of ER and mitochondria commonly known as mitochondria associated membranes (MAMs)^41^. Interestingly, it has been speculated before that SPL may be located in MAMs^3,42^.

PE synthesis in *T. brucei* (and other organisms) occurs in the ER, with the two terminal enzymes of the Kennedy pathway (TbEPT and TbCEPT) residing in different sub-compartments of the ER^43^. The mitochondrion of *T. brucei* parasites is very sensitive to changes in PE levels. Blocking PE synthesis via the Kennedy pathway by disrupting ethanolamine-phosphate cytidylyltransferase (TbET) resulted in accumulation of mitochondria with distorted morphology and ultrastructure^37,38^. Given the importance of PE for mitochondria, it is possible that TbSpl may act as a PE sensor: a drop in PE levels may result in TbSpl activation to produce ethanolamine phosphate from sphingolipid degradation for PE synthesis via the Kennedy pathway. It is surprising that depletion of TbSpl had no disruptive effect on mitochondria, even though PE levels and PE *de novo* synthesis were decreased. Instead, we observed aggregation of acidocalcisomes upon TbSpl depletion. Acidocalcisomes are conserved organelles present in both prokaryotes and eukaryotes and play a role in cation and phosphorus storage and osmoregulation^44^. The observed increase in the size of acidocalcisomes may represent an adaptation to changes in cell osmolarity in response to osmotic stress^45^. Acidocalcisomes have physical ties to mitochondria in *T. brucei*, which may be important for Ca^2+^-mediated regulation of bioenergetics^46^. It would be interesting to test if TbSpl plays a role in interorganelle communication either directly or by modulating the physical ties between acidocalcisomes and mitochondria. Moreover, recent findings have shown that acidocalcisomes are required for autophagy in *T. brucei*^47^. A possible induction of autophagy by TbSpl-depletion could be a direct consequence of increased S1P levels or be triggered indirectly by reduced PE levels. In humans, there is a direct connection between Spl and apoptosis, as the sphingolipid degradation products hexadecenal and S1P cooperate with BAK and BAX to induce apoptosis^34^.

## Material and Methods

Unless otherwise stated, all reagents were purchased from Sigma Aldrich or Merck. Restriction enzymes were purchased from Thermo Fisher Scientific (Wohlen, Switzerland). Antibiotics and fetal bovine serum (FBS) were obtained from Invitrogen (Basel, Switzerland). Radioactive L-[3–^3^H]-serine (1 mCi/ml, 20 Ci/mmol; [^3^H]-serine;) was purchased from American Radiolabeled Chemicals Inc (St. Louis, MO, USA) and dCTP [α-^32^P] (3000 Ci/mmol) was from PerkinElmer Life Sciences (Schwerzenbach, Switzerland). Primers and sequencing services were from Microsynth AG (Balgach, Switzerland).

### Trypanosomes and culture conditions

*T. brucei* procyclic form strain 29–13, co-expressing a tetracycline repressor and a T7 polymerase^48^ (obtained from Paul Englund, John Hopkins University School of Medicine), was cultured at 27 °C in SDM-79 containing 10% heat-inactivated FBS and 15 μg/ml G418 and 25 μg/ml hygromycin.

### Conditional-knockout mutants

To replace the open reading frames of TbSpl, 400 bp of the 5′ UTR and 3′ UTR flanking regions of the Tb927.6.3630 locus were amplified with primers TbSpl-KO-5 UTR-fw CCGCCTCGAGTACCGCCTTATTAGTGCCCCATGACA, TbSpl-KO-5 UTR-rv CCGCAAGCTTACTAATCACATCCTTCGTGTTTCTACTCG, TbSpl-KO-3 UTR-fw GCTCTAGAGTTGGTTATGCCGAGATGCGC, TbSpl-KO-3 UTR-rv CCGCGCGGCCGCACCAATACAATTCGTCGGCGTG (restriction sites for XhoI, HindIII and XbaI and NotI are underlined). Blasticidin and phleomycin resistance genes were inserted into plasmid pBS SK (Agilent) to produce plasmids pBS-Phleo and pBS-Blast, as described^49^. Digestion of the 5′ UTR recombination site with XhoI and HindIII and the 3′ UTR recombination site with XbaI and NotI followed by ligation into pBS-Phleo and pBS-Blast resulted in the final constructs pLDPhleKO and pLDBlastKO, respectively. Before transfections, 10 μg of plasmids were linearized with XhoI and NotI. Transfections were done as described^50^. DNA was resuspended in transfection buffer (132 mM NaCl, 8 mM KCl, 8 mM Na_2_HPO_4_, 1.5 mM KH_2_PO_4_, 0.5 mM magnesium acetate, 0.09 mM calcium acetate, pH 7.0) and transferred to a 0.2-cm pulse cuvette (Lonza, Visp, Switzerland). Electroporation was performed in 100 μl nucleocuvettes using Lonza 4D Nucleofector System (pulse code FI-115, “Primary Cell P3” solution). Stable clones were selected by limiting dilution and analyzed by PCR for correct integration of the inserts. 5 μg/mL blasticidin and 0.2 μg/mL phleomycin were used for antibiotic selection of clones, respectively.

The inducible C-terminally c-myc-tagged ectopic copy of TbSpl was constructed by PCR amplification of the ORF using primers TbSpl-c-myc-fw GCCCAAGCTTATGTCGCTGTCATGCTTTTTGGACAG and TbSpl-c-myc-rv GCGGGATCCCTAATGTTCGGTGCTGTTCGGTGCTGTAGTACTTGTTTGAG (restriction sites for HindIII and BamHI are underlined). The resulting c-myc-tagged TbSpl ORF was inserted into a pLEW100-based vector^48^ resulting in plasmid pLDSpl-c-myc, which allows tetracycline-inducible expression of c-myc-tagged TbSpl. Before transfection, the vector was linearized with NotI. Clones were obtained by limiting dilution and antibiotic selection with 2 μg/ml puromycin.

### Southern blot analysis

Southern blot analysis was done as described^49^ using 1.2 μg genomic DNA that was digested with restriction enzymes BsiWI and PaeI.

### SDS-PAGE and immunoblot analysis

Proteins were separated using 12% polyacrylamide gels according to^51^ and transferred to polyvinylidene difluoride membranes (Millipore, Billerica, MA, USA). After blocking in Tris-buffered saline (50 mM Tris-HCl, pH 7.5, 150 mM NaCl; TBS) containing 5% (w/v) milk, monoclonal mouse anti-cMyc 9E10 (1:1000; Santa Cruz Biotechnology, Heidelberg) or mouse anti eukaryotic elongation factor 1 A (1:20000; Upstate, Lake Placid, NY, USA) were used and detected using rabbit anti-mouse-HRP (Dako A/S, Glostrup, Denmark; 1:5’000) with SuperSignal West Pico (Thermo Fischer Scientific).

### Sphingosine-1-phosphate quantification

S1P was extracted according to^52^. In brief, 2 × 10^8^ cells were harvested, washed once with PBS, suspended in 1 ml PBS and spiked with 0.125 nmol (plus TbSpl) or 0.25 nmol (minus TbSpl) C17-S1P (Avanti Polar Lipids, Alabama, U.S.). After addition of 300 µl 6M HCl, 1 ml methanol and 2 ml chloroform, the mixture was thoroughly vortexed and centrifuged. The chloroform phase was retrieved and the aqueous phase re-extracted with 2 ml chloroform. The chloroform phase was dried under a stream of nitrogen and resuspended in 50 ul methanol. After 1:100 dilution with HPLC mobile phase A (methanol:water, 50:50, 1.5 mM ammonium formate, 0.2% formic acid) 5 ul were analyzed according to^53^, with minor modifications. LC-MS measurements were performed on a QExactive Plus mass spectrometer (Thermo Fisher) coupled to an EasyLC 1000 nanoflow-HPLC. HPLC-column tips (fused silica) with 75 µm inner diameter were self-packed with C8 material (3 µm, 100 Å, phenomenex) to a length of 20 cm. Analytes were separated with a gradient of A and B (2 mM ammonium formate in methanol, 0.2% formic acid) with increasing organic proportion (loading of sample with 0% B, separation ramps with a flow rate of 250 nl/min: from 5–12% B within 1 min, from 12–50% B within 9 min, from 50–100% B within 1 min, hold 100% B for 18 min). Mass spectrometer was operated in positive ion mode (ESI) with an electron spray voltage of 2.5 kV at 250 °C of the heated capillary temperature. Precursor-to-product ion transitions of m/z 380.26 → 264.27 for C18-S1P, and m/z 366.24 → 250.25 for C17-S1P were used as quantifier for parallel reaction monitoring with a normalized collision energy of 30%, resolution of 35′000, AGC target value of 200′000, and a maximum injection time of 50 ms. Relative quantification was done using Skyline Software^54^ by forming ratios between analyte peak area and internal standard peak area.

### (Immuno-) Fluorescence microscopy

MitoTracker staining and imaging was done as described^55^. Live parasites were incubated for 30 min with 0.5 $\mu$M MitoTracker Red CMXRos (Thermo Fisher Scientific) in culture medium, washed in phosphate-buffered saline (10 mM sodium phosphate, 0.15 M NaCl, pH 7.5; PBS) and let settle to microscopy slides. Parasites were fixed with 4% (w/v) paraformaldehyde, washed with PBS and mounted with Vectashield containing 4′,6-diamidino-2-phenylindole (DAPI, Vector Laboratories, CA, USA). For antibody staining, cells were permeabilized with 0.2% Triton X-100 in PBS, blocked with 2% bovine serum albumin in PBS for 30 min, and primary antibody (1:200 dilution) was added in blocking solution for 30 min. Antibodies used were rabbit anti-ATOM40 (provided by Prof. Andre Schneider, University of Bern), rabbit anti-ADP/ATP carrier (provided by Alena Ziková, Biology Centre of the Czech Academy of Sciences), rabbit anti-BiP (provided by Jay Bangs, Buffalo) and mouse anti-cMyc (Santa Cruz Biotechnology, 9E10). After washing with PBS, fluorophore-conjugated antibodies anti-rabbit Alexa Fluor 594 (Thermo Fischer Scientific) and anti-mouse Atto647 (Sigma Aldrich) were added for 30 minutes at a dilution of 1:250. After washing, cells were mounted using ProLong Diamond Antifade Mountant (Thermo Fisher Scientific) and imaged on a confocal laser scanning microscope Leica SP8 inverse STED 3×(Center for Microscopy and Image Analysis, University of Zurich) with a 93x HC PL APO STED WHITE glycerol objective with or without using the 775 nm depletion laser. Images were acquired with the Leica LAS X software (Leica Microsystems CMS GmbH, Heerbrugg, Switzerland).

### Quantification of mitochondrial membrane potential

Parasites were stained with 200 nM tetramethylrhodamine ethyl ester (TMRE) for 30 minutes at 27 °C. Control cells were treated with 25 μM carbonyl cyanide 3-chlorophenylhydrazone (CCCP) for 15 minutes prior to TMRE staining. Parasites were washed in PBS and transferred to a 96-well plate at 5 × 10^5^ cells per well. Fluorescence intensity in each well was measured with excitation at 549 nm and emission at 575 nm using a Spark microplate reader (TECAN, Männedorf, Switzerland).

### Lipid analysis

Total parasite lipids were extracted^56^ and separated by one-dimensional thin layer chromatography (TLC) on Silica Gel 60 plates (Merck, Darmstadt, Germany) in solvent system 1 composed of chloroform:methanol:acetic acid:water (25:15:4:2; v/v/v/v)^57^. Lipids were visualized by exposure to iodine vapor. For *in vivo* labeling experiments, parasites at mid-log phase were cultured in the presence of [^3^H]-serine for indicated times and lipids were extracted as above. After separation by TLC, [^3^H]-labeled lipids were analyzed by scanning the air-dried plate with a radioisotope detector (Berthold Technologies, Regensdorf, Switzerland). Quantification of radiolabeled lipids was done using the Rita Control software provided by the manufacturer. On each TLC plate, appropriate lipid standards were carried alongside. For lipid phosphorus quantification, total lipids were separated by two-dimensional TLC using solvent system 2 composed of chloroform:methanol:25% NH_3_:water (45:37:6:4; v/v/v/v) for the first dimension and solvent system 3 composed of chloroform:methanol:acetone:acetic acid:water (40:15:15:12:8; v/v/v/v) for the second dimension^57^. After exposure to iodine, lipid spots were scraped from the plates and lipid phosphorus was quantified photometrically^58^.

### Transmission electron microscopy

Parasites were washed in cold 100 mM sodium cacodylate buffer (pH 7.2) and suspended in the same buffer containing 2.5% (v/v) glutaraldehyde. Cells were fixed for 2 hours at room temperature, followed by post-fixation in 2% OsO_4_ in cacodylate buffer (pH 7.3). After three washes in distilled water, samples were pre-stained in saturated uranyl acetate solution in distilled water for 30 min at room temperature and extensively washed in distilled water. Dehydration was carried out by stepwise incubation in ethanol (30–50–70–90–100%). Parasites were then embedded in EPON812 resin as described previously^59^. Sections (80 nm) were cut on an ultramicrotome (Reichert & Jung, Vienna, Austria), placed onto 300 mesh formvar-carbon-coated nickel grids (Plano, Wetzlar, Germany), and sections were stained with uranyl acetate and lead citrate^60^. Specimens were viewed on a CM12 transmission electron microscope operating at 60 kV.

### Statistical analyses

Statistical comparison between two groups was performed using Two-way ANOVA (for TLC scanning results and lipid phosphorus determinations) done in GraphPad Prism 6.

## Supplementary information

Supplementary information.
