# Supplementary material for: Mitochondrial sphingosine-1-phosphate lyase is essential for phosphatidylethanolamine synthesis and survival of Trypanosoma brucei
Source: Sci Rep. 2020 May 19;10:8268. doi: 10.1038/s41598-020-65248-x (PMC7237492; doi:10.1038/s41598-020-65248-x)

Supplemental Material

Mitochondrial sphingosine-1-phosphate lyase is essential for phosphatidylethanolamine synthesis and survival of *Trypanosoma brucei*

Ladan Dawoody Nejad[1,2], Michael Stumpe[3], Monika Rauch[1], Andrew Hemphill[4], Roger Schneiter[3], Peter Bütikofer[1#] and Mauro Serricchio[1#]

[1]Institute of Biochemistry and Molecular Medicine, University of Bern, Bern, Switzerland
[2]Graduate School for Cellular and Biomedical Sciences, University of Bern, Bern, Switzerland
[3]Division of Biochemistry, Department of Biology, University of Fribourg, Fribourg, Switzerland
[4]Institute of Parasitology, Vetsuisse Faculty, University of Bern, Bern, Switzerland

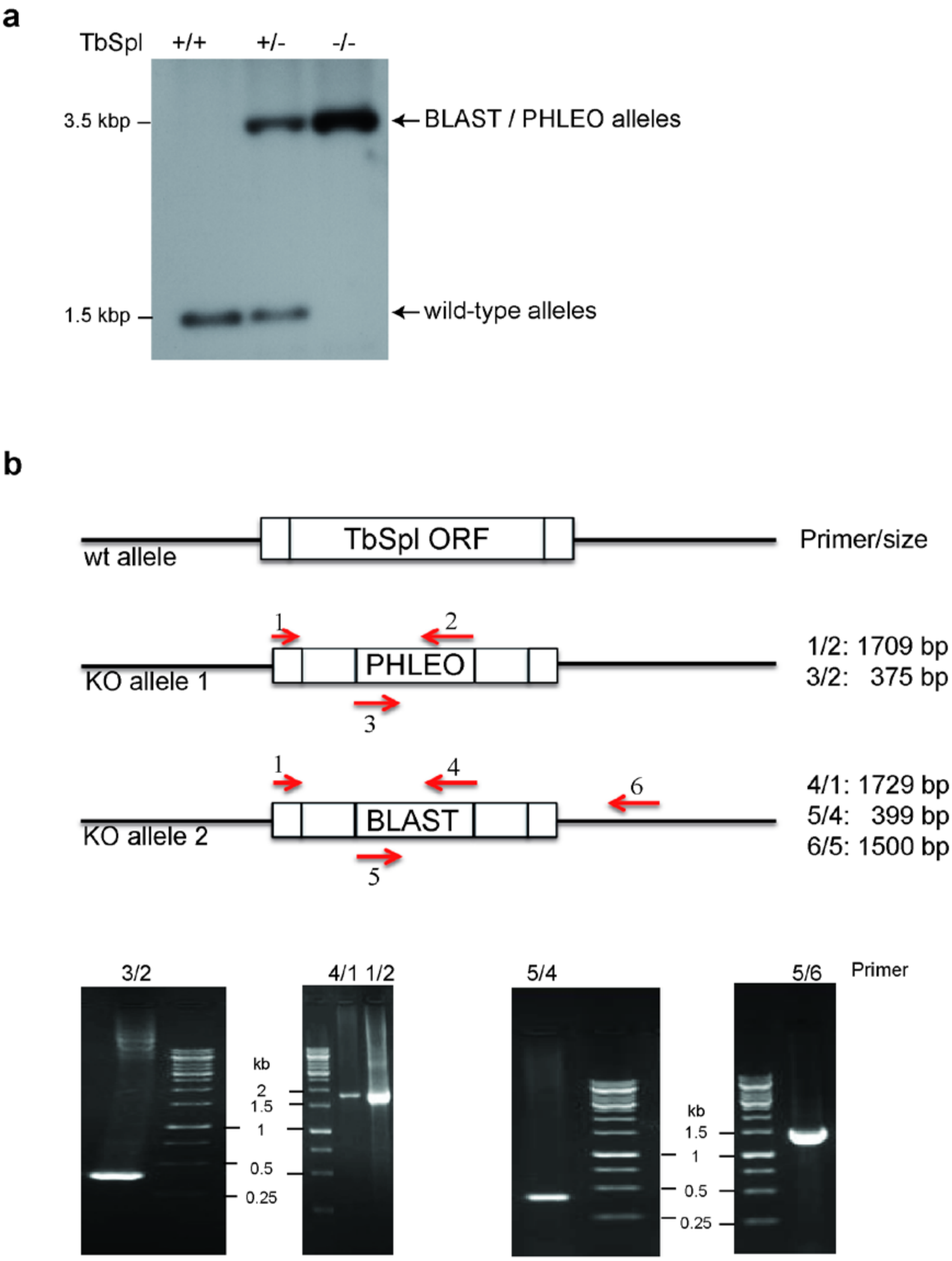

Figure S1: Verifying the TbSpl KO cell line. a) Genomic DNA of parental 29-13 *wild-type* strain (+/+), single- (+/-) and double-allele (-/-) knockout cells was extracted. After digestion of genomic DNA with PaeI and BsiWI, DNA fragments were separated by agarose gel electrophoresis, transferred onto Nitrocellulose. The membrane was analyzed with a ^{32}P-labeled probe hybridizing to the 3′ UTR of the TbSpl ORF. b) PCRs with different sets of primers were used to verify the correct integration of resistance genes into the correct loci.

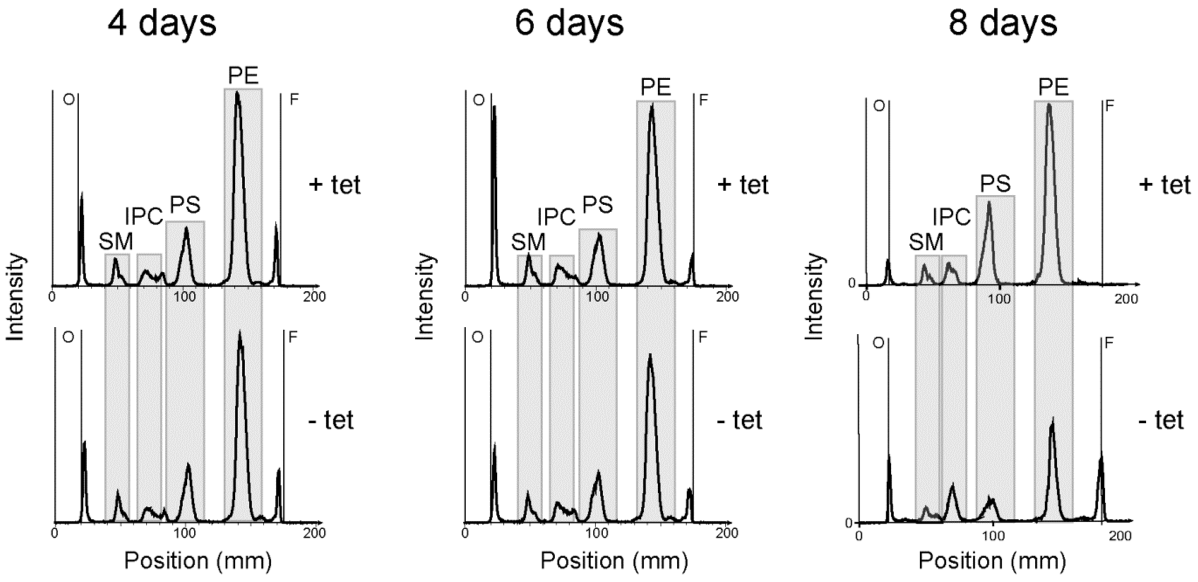

Figure S2: Metabolic labeling with ^{3}H-serine. Cells were cultured for 4, 6 or 8 days in the presence (+ tet) or absence (- tet) of tetracycline and labeled with ^{3}H-serine for the last 24 hours. Lipids were extracted and separated by thin layer chromatography and labeled lipids analyzed by radioisotope scanning. Solvent fronts (F) and origin (O) are indicated. SM: sphingomyelin; IPC: inositol phosphorylceramide; PS: phosphatidylserine; PE: phosphatidylethanolamine.

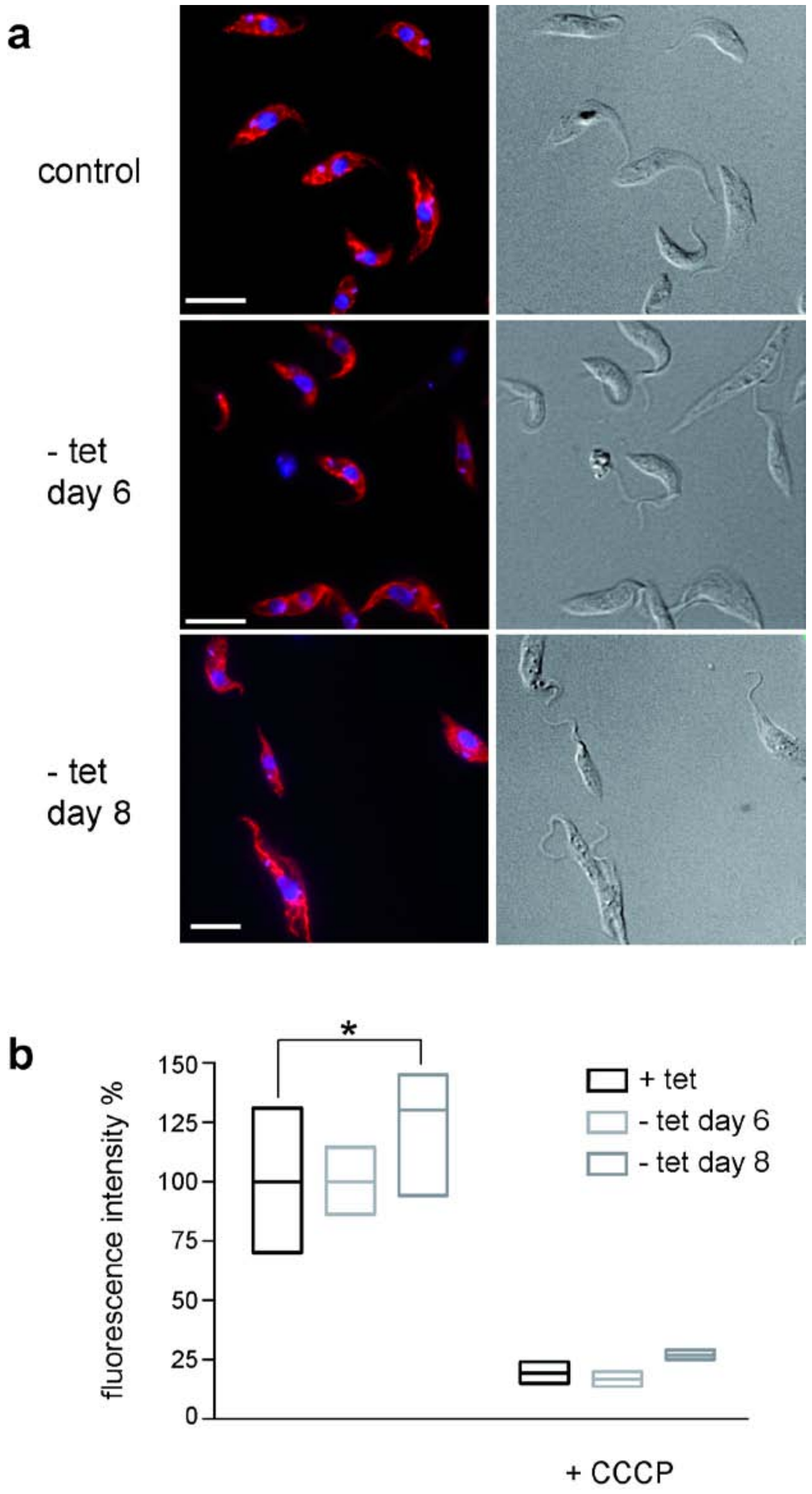

Figure S3: Assessment of mitochondrial membrane potential. a) TbSpl KO parasites were depleted of TbSpl for 0 (control), 6 or 8 days and stained with the mitochondrial membrane potential-dependent dye MitoTracker Red. Fluorescence microscopy revealed no obvious morphological defects in mitochondria or any change in MitoTracker Red intensity. Nuclei were visualized with DAPI and appear blue. b) The mitochondrial membrane potential m$\Delta\Psi$ was quantified by TMRE staining and fluorescence intensity determination using a plate reader. Carbonyl cyanide 3-chlorophenylhydrazone (CCCP) was used to dissipate the mitochondrial membrane potential. Triplicate determinations of three independent experiments are shown. Box plots with mean (horizontal line) and min-max range are shown. *: $p<0.05$

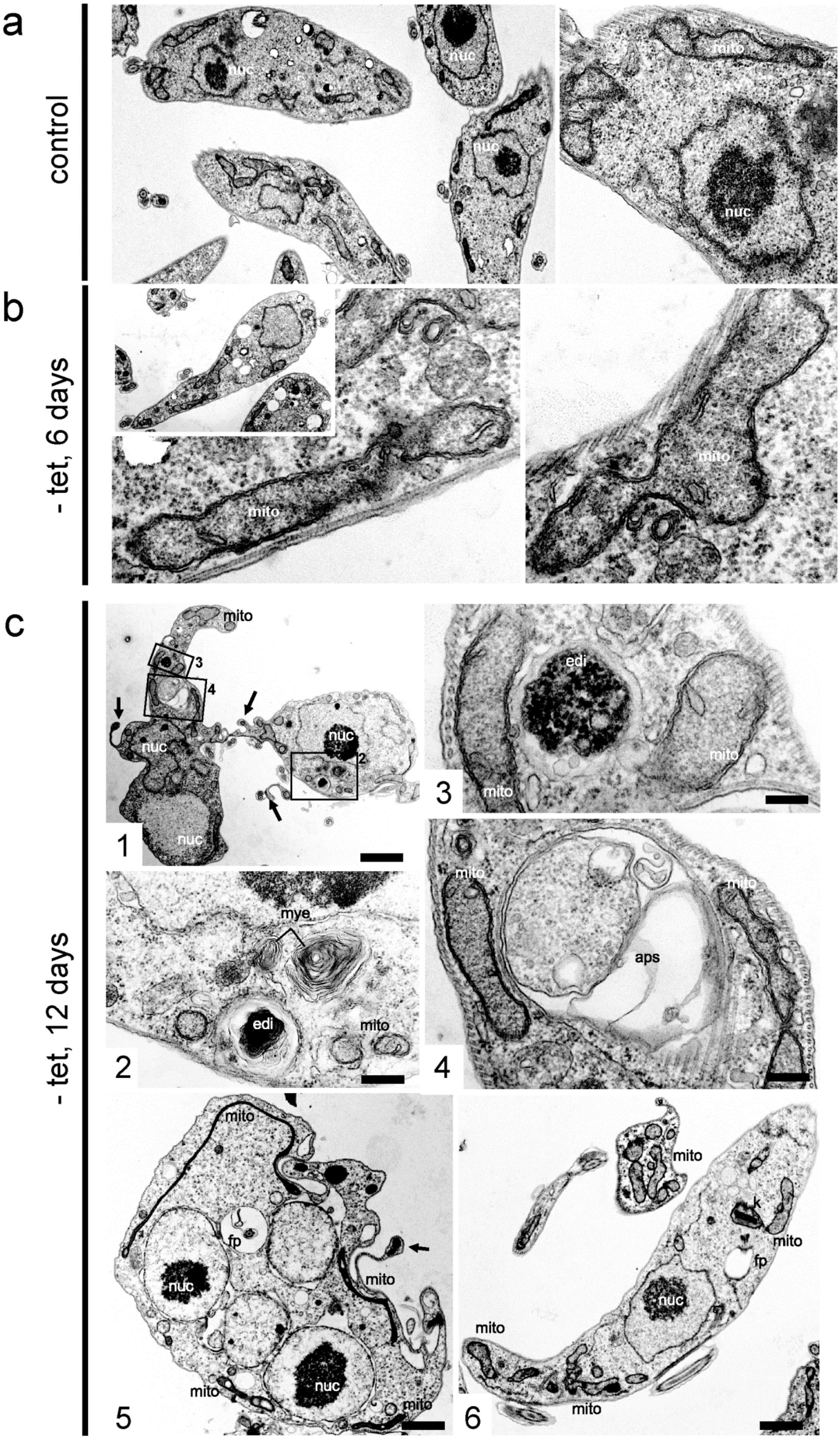

a
control
nuc
mito
b
- tet, 6 days
mito
c
- tet, 12 days
mito
3
4
nuc
2
1
edi
3
mye
aps
4
2
fp
5
k
fp
6

Figure S4: Transmission electron microscopy. a) Control and TbSpl-depleted parasites (-tet) for 6 days (b) or 12 days (c) were processed for electron microscopy. Zoomed-in regions show intact mitochondria in both control and TbSpl-depleted parasites. After 12 days of TbSpl depletion (c), 1 depicts a low magnification view (bar = 0.6 µm), and the boxed areas indicate the higher magnification views shown in 2 (bar = 0.125 µm), 3 (bar = 0.1 µm) and 4 (bar = 0.125µm). Bars in 5 and 6 = 0.6 µm. Note that after 12 days, TbSpl KO parasite cultures exhibited some structural diversity. Many parasites showed aberrant morphological and structural features including irregular surface extensions (arrows in 1 and 5), formation of myelin membrane stacks within the cytoplasm (2), cytoplasmic inclusions containing electron dense material (edi; 2, 3), and in many instances vacuoles were formed that resemble autophagosomes (aps). However, despite these alterations, mitochondria (mito) did not appear notably affected. In addition, parasites could be seen, of which the cytoplasm appeared compartmentalized, and in those instances the mitochondrial matrix was more electron dense (5), indicating the onset of cell death.
mito = mitochondrion; nuc = nucleus; edi = electron dense inclusions; aps = autophagosome-like vacuole; fp = flagellar pocket; k = kineptoplast.

Additional Figures:

Full image from Fig 1d:

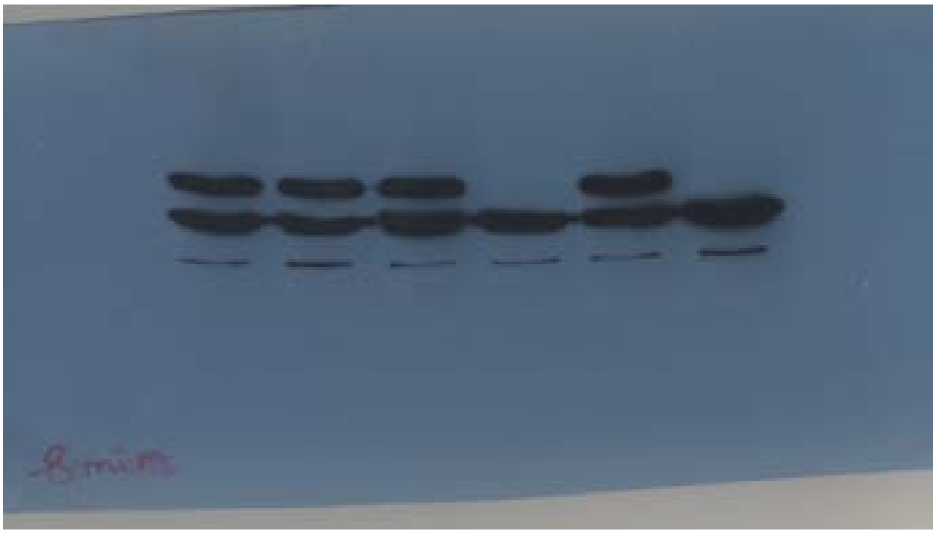

Supplement: Supplementary file 1 — Supplementary information. [file 41598_2020_65248_MOESM1_ESM.pdf]
